# Emergency Maternal Hospital Readmissions in the Postnatal Period: A Population‐Based Cohort Study

**DOI:** 10.1111/1471-0528.17955

**Published:** 2024-09-18

**Authors:** Ruth V. Pritchett, Gavin Rudge, Beck Taylor, Carole Cummins, Sara Kenyon, Ellie Jones, Sharon Morad, Christine MacArthur, Kate Jolly

**Affiliations:** ^1^ Institute of Applied Health Research University of Birmingham Birmingham UK; ^2^ Warwick Medical School University of Warwick Coventry UK; ^3^ Birmingham Women's Hospital Birmingham UK

**Keywords:** cohort, maternal, postnatal, readmission

## Abstract

**Objective:**

To determine the change in English emergency postnatal maternal readmissions 2007–2017 (pre‐COVID‐19) and the association with maternal demographics, obstetric risk factors and postnatal length of stay (LOS).

**Design:**

National cohort study.

**Setting:**

All English National Health Service hospitals.

**Population:**

A total of 6 192 140 women who gave birth in English NHS hospitals from April 2007 to March 2017.

**Methods:**

Statistical analysis using birth and readmission data from routinely collected National Hospital Episode Statistics (HES) database.

**Main Outcome Measures:**

Rate of emergency postnatal maternal hospital readmissions related to pregnancy or giving birth within 42 days postpartum, readmission diagnoses and association with maternal demographic factors, obstetric risk factors and postnatal LOS.

**Results:**

A significant increase in the rate of emergency postnatal maternal readmissions from 15 128 (2.5%) in 2008 to 20 734 (3.4%) in 2016 (aOR 1.32, 95% CI 1.28–1.37) was found. Risk factors for readmission included minoritised ethnicity (particularly Black or Black British ethnicity: aOR 1.35, 95% CI 1.31–1.39); age < 20 years (aOR 1.09, 95% CI 1.05–1.12); 40+ years (aOR 1.07, 95% CI 1.03–1.10); primiparity (multiparity: aOR 0.92, 95% CI 0.91–0.93); nonspontaneous vaginal birth modes (emergency caesarean: aOR 1.86, 95% CI 1.82–1.90); longer LOS (4+ vs. 0 days: aOR 1.58, 95% CI 1.53–1.64); and obstetric risk factors including urinary retention (aOR 2.34, 95% CI 2.06–2.53) and postnatal wound breakdown (aOR 2.01, 95% CI 1.83–2.21).

**Conclusions:**

The concerning rise in emergency maternal readmissions should be addressed from a health inequalities perspective focusing on women from minoritised ethnic groups; those < 20 and ≥ 40 years old; primiparous women; and those with specified obstetric risk factors.

## 1 Introduction

The postnatal period, from birth to 42 days postpartum [1], marks a high‐risk time for maternal death and severe morbidity [2]. Emergency (nonelective) hospital readmission is a direct indicator of maternal morbidity, which impacts women's well‐being [3, 4] and service costs and pressures [5].

Evidence suggests that the proportion of women facing emergency readmission within 42 days of giving birth in England is rising, from 2.4% in 2015 [6] to 3.3% in 2018 [7]. Factors including shifting population demographics [8], reduced provision of postnatal maternity services [9] and reductions in women's postnatal length of stay (LOS) [10] in hospital may be driving this.

Rising maternal age in many high‐income countries may be increasing medically complex pregnancies and postnatal complications [11], along with a growing proportion of births to women from minoritised ethnic groups and women born abroad [12, 13]. UK caesarean section births are also increasing, which carry an additional risk of readmission [14, 15, 16, 17].

There has been a substantial decrease in LOS in the United Kingdom, from 10 days in 1958 to 1.4 days in 2010 [18, 19, 20]. By 2017, 19.8% of women were discharged on the day of birth and 40.8% the day after [17]. The association between LOS and readmission is inconsistent worldwide [21, 22, 23]. In 2021, the UK National Institute for Health and Care Excellence (NICE) found conflicting evidence regarding the optimum time to discharge women from hospital and transfer them to community midwife care (community midwives being employed by the same maternity service provider in England) [24]. This inconsistency and the available evidence ranging from very low to moderate quality led NICE to recommend that point of discharge be determined by medical and social criteria rather than scientific research [25]. Furthermore, high‐quality research on postpartum LOS was called for [25].

In‐depth knowledge of maternal readmissions is necessary to understand and address the increase and inequalities in readmission and to guide maternity services in the provision of effective care for women at this critical time. Improving outcomes for women is vital when the safety of women's postnatal care is currently being highlighted [26].

The aims of this study were to describe the change in emergency postnatal maternal hospital readmission rates in England between 2007 and 2017 (prior to the pandemic), the causes of emergency readmissions and the association between emergency readmissions and maternal demographic factors, obstetric risk factors and postnatal LOS.

## 2 Methods

A national retrospective cohort study was conducted using National Health Service (NHS) Digital Hospital Episode Statistics (HES) data from women giving birth in all English NHS hospitals from April 2007 to March 2017. This study has been reported according to the STROBE cohort checklist [27]. In England, most women give birth in an NHS hospital, with 2.5% delivering at home [28] and 5% in private hospitals [29]. The anomalous effects of the recent COVID‐19 pandemic on maternity services may take many years to unpick and as maternity services have, in part, begun to return to their standard prepandemic operating procedures, including in‐person visits, the COVID‐19 period is not considered here.

Hospital Episode Statistics data is the minimum routinely collected dataset for all secondary care providers in England, recording hospital activity for healthcare analysis and research. Data quality varies between different fields, with small amounts of missing data reported for fields such as method of delivery (0.8%) [30] and slightly larger amounts for some maternal characteristics such as self‐reported ethnicity (8.7%) [31]. Overall, HES provides high‐quality maternal data [32].

Within English HES data from 2007 to 2017, all spells (a continuous period of time spent as a patient within a hospital or birth centre) containing a birth episode (defined by the ‘epitype’ variable and having an OPCS‐4 procedure code indicating any mode of birth) were extracted. Data included demographics, date, setting and mode of giving birth, postnatal LOS, pre‐existing and birth‐related diagnoses and operative procedures, and diagnoses and procedures relating to emergency postnatal hospital readmissions. Patient data were pseudonymised to prevent individual identification.

The primary outcome was the first emergency (nonelective) hospital readmission related to pregnancy or childbirth within 42 days of giving birth. Readmissions classified by ICD‐10 and OPCS‐4 codes (Table S1) were defined as related to pregnancy and childbirth by reference to previous literature (Table S2) and guided by experienced obstetricians and midwives. Elective hospital admissions, outpatient attendance and primary care attendance were not included. Readmission within 42 days of giving birth was used as this is the definition of the postnatal period [1]. Independent variables included maternal age, year of giving birth, ethnicity, index of multiple deprivation (IMD) income domain, parity, mode of birth, birth setting, postnatal LOS (time between birth and discharge from hospital) and maternal obstetric risk factors. Risk factors classified by ICD‐10 and OPCS‐4 codes (Table S3) were selected for their potential to influence risk of maternal readmission, by reference to existing literature (Table S4) and in consultation with obstetricians and midwives. Women were excluded from the analysis if dates of giving birth and discharge were not available.

Postnatal LOS is recorded in HES in days. A woman giving birth at 2:00 AM and discharged at 10:00 PM the same day (20 h but classed as discharge on Day 0) would have had a longer LOS than one giving birth at 11:00 PM at night and discharged at 5:00 AM the following day (6 h but classed as discharged on Day 1). Despite this unavoidable disparity, mean postnatal LOS days still provides a consistent indication of the direction of any effect.

### 2.1 Statistical Analysis

The incidence of emergency postnatal maternal hospital readmissions related to pregnancy or childbirth 2007–2017 within 42 days of birth and diagnoses associated with readmissions were calculated. Demographic and obstetric characteristics of women not readmitted and readmitted within 42 days were described as well as changes in demographics over time.

The variance inflation factor (VIF) for each variable to be included in a regression model was calculated to identify multicollinearity. Highly associated variables with a VIF > 10 were removed. A backward stepwise logistic regression using adjusted odds ratios was then undertaken to create a model of demographic and obstetric factors associated with emergency postnatal maternal readmission. Cluster robust standard errors for women were used to account for the nonindependence of data from women giving birth on more than one occasion within the dataset. Independent factors included age, year of giving birth, ethnicity, IMD income domain, parity, mode and setting of birth, postnatal LOS and maternal obstetric risk factors. Factors without a significant association with emergency maternal readmission were removed sequentially from the model; the factor with the highest *p*‐value being removed first and at every further iteration. Odds ratios were adjusted for all demographic and obstetric factors. Analyses were conducted in STATA 17 [33].

## 3 Results

We identified 6 206 638 women who gave birth in English NHS hospitals from April 2007 to March 2017. The following individuals were excluded from the analysis: 4921 due to implausibly short intervals between two separate births from the same mother (most likely errors in patient identity); 3673 due to implausible admission dates or dates commencing before the study period; 2233 due to missing discharge dates; 3619 duplicate spells; 47 cases wrongly coded as maternal that were in fact infant‐related; and five cases of women of implausibly young age (0–11 years). The final analysis included 6 192 140 women (Table S5).

### 3.1 Emergency Readmission for Conditions Relating to Pregnancy and Childbirth

There were 297 008 (4.8%) emergency postnatal maternal hospital readmissions within 42 days of giving birth, 180 309 (60.7%) of which were related to pregnancy or giving birth (2.9% of all women giving birth). A consistent annual increase in the proportion of emergency postnatal maternal readmissions relating to pregnancy and childbirth occurred during this decade from 2.5% (15 128) in 2008 to 3.4% (20 734) in 2016. In 2016, 5606 more women were readmitted than in 2008 (years with data from all 12 months) (Table 1).

**TABLE 1:** Emergency maternal readmissions between 2007 and 2017.

Year of delivery	Number of mothers giving birth	Emergency readmission within 42 days of birth (*n*, % of mothers giving birth)	Emergency readmission within 42 days of birth, related to pregnancy or childbirth (*n*, % of mothers giving birth)
2007	451 625	19 134 (4.2)	10 912 (2.4)
2008	607 747	26 483 (4.4)	15 128 (2.5)
2009	611 946	27 657 (4.5)	15 869 (2.6)
2010	637 337	28 762 (4.5)	16 928 (2.7)
2011	637 740	30 163 (4.7)	18 215 (2.9)
2012	647 430	30 921 (4.8)	18 563 (2.9)
2013	622 576	30 974 (5.0)	19 199 (3.1)
2014	614 350	31 305 (5.1)	20 001 (3.3)
2015	613 880	31 423 (5.1)	19 845 (3.2)
2016	608 360	32 568 (5.4)	20 734 (3.4)
2017	139 149	7618 (5.5)	4914 (3.5)
Total	6 192 140	297 008 (4.8)	180 309 (2.9)

### 3.2 Readmission Diagnoses Relating to Pregnancy and Childbirth

The most common specific readmission diagnosis was puerperal infection (33 759, 18.0%), followed by haemorrhage (27 145, 14.5%); hypertension‐related complications (15 728, 8.4%); postnatal wound breakdown (13 756, 7.3%); puerperal sepsis (8756, 4.7%); mastitis (6319, 3.4%); spinal or epidural anaesthesia headache and other complications of anaesthesia (4667, 2.5%); anaemia with transfusion (4087, 2.2%); diseases of the circulatory system complicating pregnancy, birth and the puerperium (3257, 1.7%); urinary retention (2902, 1.5%); and urinary tract infection (2069, 1.1%) (Table 2). The largest group of readmissions were categorised as ‘other conditions relating to the perinatal period’ (49 650 women, 26.5%). This combined category included ‘other specified diseases and conditions complicating pregnancy, childbirth and the puerperium’ (ICD‐10 code O99.8) and women with an ‘O’ ICD‐10 code from a readmission spell indicating a maternal‐related diagnosis, where the diagnosis itself was unclassified or did not fit within a specific diagnostic category. For further conditions resulting in ≤ 1% of emergency postnatal maternal readmissions, see Table 2.

**TABLE 2:** Emergency readmissions related to pregnancy or childbirth within 42 days of giving birth.

Readmission diagnosis (a primary diagnosis recorded within the readmission spell)	Emergency postpartum readmissions within 42 days of birth, related to pregnancy or childbirth (*N*, % of all readmissions within 42 days 187 327)
Other conditions relating to the perinatal period	49 650 (26.5)
Puerperal infections, other	33 759 (18.0)
Haemorrhage	27 145 (14.5)
Hypertension‐related complications	15 728 (8.4)
Postnatal wound breakdown	13 756 (7.3)
Puerperal sepsis	8756 (4.7)
Mastitis	6319 (3.4)
Spinal or epidural anaesthesia headache and other complications of anaesthesia	4667 (2.5)
Anaemia	4087 (2.2)
Diseases of the circulatory system complicating pregnancy, birth and the puerperium	3257 (1.7)
Urinary retention	2902 (1.5)
Urinary tract infection	2069 (1.1)
Diseases of the digestive system complicating pregnancy, birth and the puerperium	1925 (1.0)
Retained products of conception without haemorrhage	1910 (1.0)
Breast inflammation	1864 (1.0)
Venous thromboembolism	1380 (0.7)
Lactation issues	1356 (0.7)
Perineal laceration	1107 (0.6)
Medical misadventure	967 (0.5)
Diseases of the respiratory system complicating pregnancy, birth and the puerperium	912 (0.5)
Postpartum psychosis	639 (0.3)
Other mental health conditions	507 (0.3)
Obstetric trauma, other	454 (0.2)
Diseases of the skin and subcutaneous tissue complicating pregnancy, birth and the puerperium	397 (0.2)
Inflammatory diseases of the uterus	347 (0.2)
Cardiomyopathy in the puerperium	232 (0.1)
Anxiety	212 (0.1)
Gestational diabetes	174 (0.1)
Bipolar affective disorders	157 (0.1)
Liver disorders in pregnancy, birth or the puerperium	122 (0.1)
Depression	110 (0.1)
Difficulty establishing bowel function	110 (0.1)
Total number of pregnancy/childbirth‐related readmissions (% of all maternal readmissions in this timeframe 297 008)	187 327 (63.1)

### 3.3 Postnatal Length of Stay

Between April 2007 and March 2017, 10.1% (628 017) of women were discharged on the day of giving birth; 31.6% (1 955 156) the day after; 25.4% (1 575 177) 2 days after; 14.2% (877 445) 3 days after; 18.7% (1 158 345) 4 or more days after. Between 2007 and 2017 there was no consistent trend in the proportion of women discharged on the day of giving birth (Table S6). For variation in maternal characteristics by LOS, see Table S6.

### 3.4 Demographic and Birthing Risk Factors

A significant increase in the likelihood of emergency readmission was seen each year from 2008 to 2017 (aOR 1.38, 95% CI 1.32–1.44) (Table 3). Maternal age < 20 (aOR 1.09, 95% CI 1.05–1.12) and 40+ years (aOR 1.07, 95% CI 1.03–1.10) were associated with an increased risk of emergency readmission compared to 20–24 years (Table 3). Compared to women of White or White British ethnicity, women of Black or Black British ethnicity (aOR 1.35, 95% CI 1.31–1.39), Asian or Asian British ethnicity (aOR 1.08, 95% CI 1.06–1.10), mixed ethnicity (aOR 1.10, 95% CI 1.05–1.16) and other ethnicity (aOR 1.08, 95% CI 1.04–1.12) were significantly more likely to be readmitted. It is notable that mean income deprivation was higher among Black or Black British women (0.26, SD 0.12) and Asian or Asian British women (0.24, SD 0.13) than White or White British women (0.16, SD 0.11) (Table S7); however, all levels of deprivation below the highest‐income category carried a similar association with readmission (Table 3). Multiparous women were significantly less likely to be readmitted compared to primipara (aOR 0.92, 95% CI 0.91–0.93). Compared to spontaneous vaginal birth, all other birth modes were significantly associated with higher risk of emergency readmission, with emergency caesarean section carrying the highest risk (aOR 1.86, 95% CI 1.82–1.90). ‘Professional in charge of the birth’ was removed from the model due to being highly associated with birth setting (consultant‐led ward, midwife‐led ward, etc.) (VIF > 10). Giving birth in a hospital midwife‐led ward (also called alongside midwife units, midwifery units or birth centres, these are units run by midwives where low‐risk women can choose to give birth) was associated with a slightly higher risk of readmission compared to a hospital consultant‐led ward (aOR 1.08, 95% CI 1.06–1.11). Compared to women discharged on the day of giving birth, women discharged on subsequent days had a significantly higher risk of emergency readmission: day after birth (aOR 1.16, 95% CI 1.12–1.19); 2 days (aOR 1.42, 95% CI 1.38–1.46); 3 days (aOR 1.60, 95% CI 1.55–1.65); and 4 or more days (aOR 1.58, 95% CI 1.53–1.64) (Table 3). For a full breakdown of maternal characteristics, see Table S8. For details of how women's demographics changed over the course of the study, including a small decrease in the proportion of White British women (420 382, 77.7% in 2008; 421 719, 77.3% in 2016), an increase in maternal age (mean 28.9, SD 6.1 in 2008; mean 29.8, SD 5.6 in 2016), a decrease in the proportion of spontaneous vaginal deliveries (381 741, 63.1% in 2008; 363 478, 59.8% in 2016) and increased levels of risk factors such as postpartum haemorrhage (60 860, 10.0% in 2008; 106 653 17.5% in 2016) and mental health conditions (7154, 1.2% in 2008; 42 051, 6.9% in 2016), see Table S9. For unadjusted odds ratios for all demographic and birthing factors, see Table S10.

**TABLE 3:** Demographic and birth factors associated with emergency maternal readmission ≤ 42 days after birth relating to pregnancy or childbirth: final modal after backwards stepwise logistic regression.

	Adjusted odds ratio	95% CI		*N*
**Age group**				
<20 years	1.09	1.05	1.12	301 644
20–24 years (comparator)				1 096 955
25–29 years	0.96	0.94	0.97	1 717 639
30–34 years	0.98	0.96	1.00	1 792 166
35–39 years	1.00	0.98	1.02	911 683
40+ years	1.07	1.03	1.10	234 157
**Year of delivery**				
2007	0.98	0.94	1.03	451 625
2008 (comparator as 2007 is not a full data year)				607 747
2009	1.03	1.00	1.06	611 946
2010	1.06	1.03	1.10	637 337
2011	1.12	1.08	1.15	637 740
2012	1.11	1.08	1.15	647 430
2013	1.20	1.16	1.24	622 576
2014	1.27	1.23	1.32	614 350
2015	1.24	1.21	1.28	613 880
2016	1.32	1.28	1.37	608 360
2017	1.38	1.32	1.44	139 149
**Ethnicity**				
White/White British (comparator)				4 373 558
Asian/Asian British	1.08	1.06	1.10	682 725
Black/Black British	1.35	1.31	1.39	304 063
Mixed	1.10	1.05	1.16	94 586
Other	1.08	1.04	1.12	179 299
**Income domain quintile of the index of multiple deprivation**				
1 (Comparator, highest income)				1 734 563
2	1.07	1.05	1.09	1 370 437
3	1.11	1.08	1.13	1 136 986
4	1.09	1.07	1.11	971 318
5	1.10	1.08	1.12	876 978
**Parity**				
Primiparity (comparator)				1 600 857
Multiparity	0.92	0.91	0.93	2 536 898
**Delivery method**				
Spontaneous vaginal delivery (comparator)				3 802 535
Operative vaginal delivery	1.62	1.59	1.66	784 721
Breech vaginal delivery	1.21	1.10	1.33	26 001
Elective caesarean section	1.56	1.51	1.61	629 688
Emergency or other type of caesarean section	1.86	1.82	1.90	932 131
Other^a^	2.37	1.48	3.80	554
**Place of delivery**				
NHS hospital: delivery facilities with consultant ward (comparator)				2 474 142
NHS hospital: delivery facilities with midwife ward	1.08	1.06	1.11	617 575
NHS hospital: delivery facilities with GP ward	0.71	0.63	0.80	31 105
NHS hospital: delivery facilities with two of consultant/GP/midwife ward	1.07	1.05	1.08	1 875 303
NHS hospital: ward/unit without delivery facilities	1.06	0.92	1.22	7922
Other^b^	0.83	0.76	0.90	48 219
**Length of postnatal hospital stay**				
Discharged on day of birth (comparator)				628 017
Discharged day after birth	1.16	1.12	1.19	1 955 156
Discharged 2 days after birth	1.42	1.38	1.46	1 573 177
Discharged 3 days after birth	1.60	1.55	1.65	877 445
Discharged 4 or more days after birth	1.58	1.53	1.64	1 158 345

*Note:* This model is fully adjusted for all the above factors and all obstetric risk factors from Table 4. Missing data by variable (from a total of 6 192 140): age group 57 896, 0.9%; year of giving birth 0, 0%; ethnicity 557 909, 9.0%; income domain of the index of multiple deprivation score 101 858, 1.6%; parity 2 054 385, 33.2%; delivery method 16 510, 0.3%; delivery setting 1 137 874, 18.4%.

^a^ Destructive operation to facilitate delivery, other specified or other unspecified delivery method.

^b^ Including private hospital, domestic address followed by admission to hospital, other institution and other setting.

### 3.5 Obstetric Risk Factors

Women with the following conditions in pregnancy or during the birth spell had a significantly higher risk of emergency readmission: urinary retention (aOR 2.34, 95% CI 2.06–2.53), postnatal wound breakdown (aOR 2.01, 95% CI 1.83–2.21), venous thromboembolism (aOR 1.88, 95% CI 1.60–2.20), pre‐eclampsia (aOR1.88, 95% CI 1.82–1.95), stillbirth (aOR 1.87, 95% CI 1.74–2.01), other hypertensive conditions (aOR 1.79, 95% CI 1.74–1.85), gestational hypertension (aOR 1.79, 95% CI 1.74–1.85), medical misadventure (aOR 1.60, 95% CI 1.34–1.91), eclampsia (aOR 1.54, 95% CI 1.29–1.84), postpartum haemorrhage (aOR 1.40, 95% CI 1.36–1.40), pre‐existing Lupus (aOR 1.37, 95% CI 1.12–1.69), retained products of conception (aOR 1.36, 95% CI 1.27–1.45), mental health conditions (aOR 1.28, 95% CI 1.25–1.32) and preterm birth (aOR 1.28, 95% CI 1.24–1.33) (Table 4). For obstetric risk factors associated with readmission with an aOR below 1.2, see Table 4.

**TABLE 4:** Obstetric risk factors associated with emergency maternal readmission ≤ 42 days after birth relating to pregnancy or childbirth: final modal after backwards stepwise logistic regression.

Obstetric risk factors	Adjusted odds ratio	95% CI		*N*
Urinary retention	2.34	2.16	2.53	12 630
Postnatal wound breakdown	2.01	1.83	2.21	9309
Venous thromboembolism	1.88	1.60	2.20	4690
Preeclampsia	1.88	1.82	1.95	122 260
Stillbirth	1.87	1.74	2.01	31 002
Other hypertension	1.79	1.74	1.85	129 970
Gestational hypertension	1.79	1.74	1.85	140 654
Medical misadventure	1.60	1.34	1.91	3271
Eclampsia	1.54	1.29	1.84	3693
Postpartum haemorrhage	1.40	1.36	1.40	827 310
Pre‐existing lupus	1.37	1.12	1.69	3221
Retained products of conception	1.36	1.27	1.45	49 117
Mental health conditions	1.28	1.25	1.32	209 174
Preterm delivery	1.28	1.24	1.33	498 203
Other maternal factors not elsewhere categorised	1.17	1.14	1.20	362 875
Pre‐existing heart disease	1.15	1.00	1.31	9509
Intrapartum haemorrhage	1.14	1.05	1.25	24 935
Pre‐existing Type 2 diabetes	1.12	1.02	1.24	14 709
Pre‐existing asthma	1.11	1.08	1.14	363 403
Anaemia with transfusion	1.11	1.02	1.21	25 867
Other obstetric trauma	1.10	1.06	1.14	198 999
Other puerperal infection	1.10	1.05	1.16	65 289
Polyhydramnios	1.09	1.04	1.15	64 711
Social factors	1.09	1.01	1.17	41 704
Previous caesarean	1.06	1.03	1.08	635 257
Gestational diabetes	1.06	1.03	1.09	225 680
Antepartum haemorrhage	1.06	1.01	1.11	91 984
Perineal laceration	1.05	1.04	1.07	2 513 690
Failed induction of labour	1.05	1.02	1.07	708 039
Smoking	0.97	0.95	0.99	548 295
Premature rupture of membranes	0.96	0.94	0.98	622 634
Puerperal sepsis	0.91	0.87	0.94	352 144
Poor foetal growth	0.89	0.86	0.92	199 832
Drug use or dependence	0.75	0.67	0.84	21 352

*Note:* This model is fully adjusted for all the above factors and all demographic and birth factors from Table 3.

Women with the following conditions in pregnancy or during the birth spell had a significantly decreased risk of emergency readmission: being a smoker (aOR 0.97, 95% CI 0.95–0.99), premature rupture of membranes (aOR 0.96, 95% CI 0.94–0.98), puerperal sepsis (aOR 0.91, 95% CI 0.87–0.94), poor foetal growth (aOR 0.89, 95% CI 0.86–0.92) and drug use or dependence (aOR 0.75, 95% CI 0.67–0.84). For full details of obstetric risk factors not significantly associated with risk of readmission, see Table S11. For unadjusted odds ratios for all obstetric risk factors, see Table S10.

## 4 Discussion

### 4.1 Main Findings

Emergency postnatal maternal readmissions in England related to pregnancy and childbirth increased significantly over the prepandemic decade, from 2.4% in 2007 to 3.5% in 2017, representing an additional 5606 women readmitted annually by the end of the decade. The most common reasons for emergency readmissions were infection, haemorrhage, hypertension, wound breakdown, spinal and epidural anaesthesia headache, anaemia and urinary retention. Relative to discharge on day of giving birth, longer postnatal hospital stays were associated with greater risk of emergency readmission. Maternal factors significantly associated with increased risk of emergency readmission included maternal age < 20 and 40+ years, being from a minoritised ethnic group (in particular women of Black or Black British ethnicity), primipara women, delivering by emergency or elective caesarean section, operative vaginal birth and vaginal breech birth. Giving birth in a hospital midwife‐led ward was associated with a slightly higher risk of readmission compared to a consultant‐led ward. A wide range of obstetric risk factors were associated with an increased likelihood of readmission, including urinary retention, postnatal wound breakdown, venous thromboembolism, hypertensive conditions, stillbirth, medical misadventure and postpartum haemorrhage. Several obstetric factors decreased the risk of readmission including smoking, premature rupture of membranes, puerperal sepsis, poor foetal growth and drug use.

### 4.2 Strengths and Limitations

This research is the largest and most up‐to‐date study of maternal postnatal emergency hospital readmissions in England. Our population‐based findings include the vast majority of women giving birth in English NHS hospitals from April 2007 to March 2017, with few exclusions. High‐quality HES data were utilised. Detailed input from obstetricians and midwives informed the relevance and completeness of our clinical outcomes and maternal obstetric risk factors.

LOS is recorded in days rather than hours in HES resulting in some overlap between discharge categories (0, 1, 2, 3 and 4 or more days after birth) in terms of hours spent in hospital. This may decrease our findings' precision, although overall trends would be unaffected. Data on home births (2.5% of English births [28]) and women who delivered in private hospitals (5% of English births [29]) were not available for comparison.

This research does not consider the COVID‐19 pandemic or immediate postpandemic period as the profound effect of the pandemic on maternity care would have artificially altered any trends in postnatal maternal readmissions.

### 4.3 Interpretation

This research has provided detail on a concerning increase in emergency postnatal maternal readmissions in England over the decade studied. Such an increase has also been reported in other high‐income countries such as the United States [34], from 1.72% in 2004 to 2.16% in 2011 in California, Florida and New York to 6.3% in 2017 across the United States [35]. Various factors may be driving this increase, including a rise in the proportion of women from minoritised ethnic groups, increasing maternal age, a decreasing proportion of spontaneous vaginal births and a rise in risk factors for readmission such as postpartum haemorrhage and mental health conditions.

Significantly higher readmission was found among women from minoritised ethnic groups, especially women of Black or Black British ethnicity. Women of Black or Black British ethnicity were found to live in areas with the highest levels of income deprivation; however, all quintiles below the highest‐income category had a similar risk of readmission suggesting deprivation was not the underlying factor. Women from minoritised ethnic groups have been found to have higher levels of maternal morbidity [13] and mortality [36]. Poorer health during pregnancy and birth may be influencing their risk of readmission in the postpartum period, although this analysis accounted for many common comorbidities of pregnancy. A more proactive approach to rigorously evaluating and implementing effective interventions focusing on the health of pregnant women from minoritised ethnic groups within maternity services and public health is needed [37].

All modes of birth other than spontaneous vaginal birth incurred a higher risk of readmission, with emergency caesarean section carrying a greater risk of readmission than elective. The rise in rates of emergency caesarean section in England in recent years from 15% in 2015 to 22% in 2023 [38, 39] may explain some of the increase in readmission. This pattern of increasing numbers of caesarean sections has not been uniform across European countries [40]. Insights from countries such as Norway and The Netherlands which have maintained relatively low levels of caesarean sections may provide useful insights [40].

Women giving birth in hospital midwife‐led wards were at higher risk of readmission than those giving birth in a consultant‐led ward. This finding contrasts with previous research that found midwife‐led settings reduced intervention rates in low‐risk women [41]. NICE recommends that women with obstetric risk factors should be informed of the risks and benefits of giving birth in midwife‐led compared to consultant‐led settings [42]. Further research is needed to explore women's birth setting choices and whether they are fully informed [43].

Our findings support a policy of discharge on day of birth in low‐risk women, with no increased risk of emergency postnatal maternal readmission identified. Shorter postnatal LOS appears to be being offered appropriately, providing the evidence called for by NICE to support the recommendations within its recent guidelines [24]. The effect of shorter postnatal LOS on primary care attendances, midwife and health visitor contacts and breastfeeding is not known.

Given the dominance of infectious and wound‐related complications after birth, prevention of infectious complications should be a focus of maternal postnatal care. Our findings highlight the importance of full implementation of recent World Health Organisation (WHO) guidance advising routine antibiotic prophylaxis for women undergoing operative vaginal birth and the potential of the newly developed WHO postpartum haemorrhage care bundles in reducing readmissions [44, 45, 46]. Despite the current pressures within community postnatal care services [47], our findings also emphasise the importance of discussion and, where indicated, examination of perineal and caesarean section wounds by midwives in the early postnatal period. Our findings also call into question the balance between the current widespread use of anticoagulants for prevention of venous thromboembolism and the high prevalence of haemorrhagic and wound breakdown‐related readmissions [48].

Hypertensive conditions and urinary retention were also key reasons for readmission and should be paid particular attention by midwives in the immediate postnatal period and GPs in the following weeks so that urgent care can be received when needed. The readmission of women for mastitis may also be a signal for improved breastfeeding support and breast care [49]. Readmissions for debilitating spinal or epidural anaesthesia‐related headaches, caused by the unintentional puncturing of the spinal dura, were also substantial, highlighting the need for preventative measures and suitable care for such women [50].

Several conditions have been highlighted that, perhaps surprisingly, decreased the risk of readmission. A lower risk of readmission associated with sepsis during the birth or postnatal hospital stay was noted. The high‐profile Surviving Sepsis Campaign and guidelines, which were initiated in the years before this study [51], effective following the Royal College of Obstetricians and Gynaecologists Bacterial Sepsis in Pregnancy Guideline [52] and judicious use of Maternal Sepsis Pathways in NHS hospitals [53] may have supported the successful identification and treatment of sepsis, preventing future emergency readmissions to a greater degree than for other puerperal infections. Similarly, the lower risk of readmission among women experiencing premature rupture of membranes may indicate that induction and prophylactic antibiotics are being successfully offered and are preventing potential readmissions for conditions such as chorioamnionitis [54].

Factors such as smoking and drug use are known to be associated with poor foetal growth [55, 56]. Smoking, drug use and poor foetal growth were all associated with a lower risk of readmission. It may be that a lack of care‐seeking behaviour, often found among people using restricted substances, may be resulting in poor engagement with health services after giving birth [57].

Reduced provision of community postnatal care from midwives and health visitors could also be driving increased readmissions, with fewer opportunities to detect problems [9, 58]. Recently, questions have also been raised about the knowledge and training of GPs in postnatal care [59]. Women have indicated that they would prefer more postnatal home visits, leading to dissatisfaction with community care [60]. In light of the rise in emergency readmissions and the increased obstetric complexity of births, it is concerning that women are being offered reduced home‐based postnatal care.

## 5 Conclusions

The rise in emergency postnatal maternal readmissions over the prepandemic decade and the reasons for these readmissions, explored by this research, are concerning. Our research has highlighted which women are at increased risk of readmission, enabling consideration of interventions to detect problems early and prevent readmission. Greater focus is required on the health of younger (< 20) and older (40+) pregnant women, primiparae, those with certain obstetric risk factors and those giving birth by caesarean section or operative vaginal delivery. Health inequalities in readmission, particularly the high risk among women from minoritised ethnic groups, most notably those from a Black or Black British background, need to be addressed. However, shorter postnatal LOS, such as discharge on day of birth, does seem to be being offered appropriately, without any increase in risk of readmission.

From a research perspective, our findings have highlighted evidence around the slightly higher readmission of women cared for in midwife‐led wards. Research evaluating the use of different birth settings for a range of women specifically with regard to readmission, rather than interventions or setting transfers would be beneficial. From a systems perspective, the decrease in hospital‐ and community‐based midwifery provision in the United Kingdom and the training needs of GPs also require focus if opportunities to identify maternal health problems and prevent emergency readmission are not to be missed.

## Author Contributions

R.V.P. and G.R. were involved in the conception, planning, carrying out, analysing and writing up of the study; and C.M., B.T., K.J., C.C., S.K., E.J. and S.M. were involved in the conception and planning of the study, interpreting results and editing the paper.

## Ethics Statement

Ethical approval and sponsorship of this research were provided by the University of Birmingham (reference ERN_19–1234).

## Conflicts of Interest

RVP, GR, KJ, CC, SK, SM and CM declare no conflicts of interest. BT reports being a co‐investigator on a range of NIHR grants and a Medical Research Council Grant. EJ reports having an NIHR Advanced Fellowship.

## Supporting information


Table S1.

## Data Availability

The data that support the findings of this study are available from NHS Digital. Restrictions apply to the availability of these data, which were used under license for this study. Data are available from the author(s) with the permission of NHS Digital.
