# Supplementary material for: Emergency Maternal Hospital Readmissions in the Postnatal Period: A Population‐Based Cohort Study
Source: BJOG. 2024 Sep 18;132(2):178–88. doi: 10.1111/1471-0528.17955 (PMC11625651; doi:10.1111/1471-0528.17955)
Supplement: Supplementary file 1 — Table S1. [file BJO-132-178-s001.zip › bjo17955-sup-0001-TableS1.docx]

**Supplementary Table 1: ICD-10 and OPCS-4 coding for obstetric risk factors and readmission diagnoses**

|  | **Condition** | **ICD-10 coding** |
| --- | --- | --- |
| **Readmission diagnoses recorded in the readmission episode** | Other conditions relating to the perinatal period | O99.8 and O10-O75 or O85-O99 but not already categorised |
|  | Puerperal infections, other | O86, O75.3, T81.4, O41.1, O75.2, O75.3 |
|  | Haemorrhage | O72, N93.9, O46.9, O20.9 |
|  | Hypertension related complications | O10, O11, O13-O16 |
|  | Postnatal wound breakdown | O90.0, O90.1, O90.2, O34.2 |
|  | Puerperal sepsis | O85 |
|  | Mastitis | O91 |
|  | Spinal or epidural anaesthesia headache and other complications of anaesthesia | O74.5, O29.4, O89.8, O74.8, O74.9, O29.5, O89.9, O74.3 |
|  | Anaemia with transfusion | O99.0, D64.9 and one of: Z51.3, X33.2, X33.3, X33.8, X33.9, X34 |
|  | Diseases of the circulatory system complicating pregnancy, birth and the puerperium | O99.4, O87.0, O87.2, O87.9, O87.8, O99.1, O22.2, O22.4 |
|  | Urinary retention | R33, O90.4 |
|  | Diseases of the digestive system complicating pregnancy, birth and the puerperium | O99.6 |
|  | Urinary tract infection | N10-N12, N23, N30 |
|  | Retained products of conception without haemorrhage | O73.0, O73.1 |
|  | Breast inflammation | N61 |
|  | Venous thromboembolism | I26.9, I80.1, I80.2, I81, I82.2, I82.3, I63.6, I67.6, O87.1, O87.3, O88.2, O22.3, O22.5 |
|  | Lactation issues | O92 |
|  | Perineal laceration | O70 |
|  | Medical misadventure | F60-F66, F69, Y70-Y84 |
|  | Diseases of the respiratory system complicating pregnancy, birth and the puerperium | O99.5, O98.0 |
|  | Postpartum psychosis | F20, F22-F25, F28, F29 |
|  | Other mental health conditions | O99.3, F53.9, F39, F53.0, F53.1, F53.8 |
|  | Obstetric trauma, other | O71, N35.0, R31.1, R32.2 |
|  | Diseases of the skin and subcutaneous tissue complicating pregnancy, birth and the puerperium | O99.7 |
|  | Inflammatory diseases of the uterus | N71 |
|  | Cardiomyopathy in the puerperium | O90.3 |
|  | Anxiety | F40-F45, F48 |
|  | Gestational diabetes | O24.4, O24.9 |
|  | Bipolar affective disorders | F30, F31 |
|  | Liver disorders in pregnancy, birth or the puerperium | O26.6 |
|  | Depression | F32, F33, |
|  | Difficulty establishing bowel function | K59.0, R15 |
