# Supplementary material for: Emergency Maternal Hospital Readmissions in the Postnatal Period: A Population‐Based Cohort Study
Source: BJOG. 2024 Sep 18;132(2):178–88. doi: 10.1111/1471-0528.17955 (PMC11625651; doi:10.1111/1471-0528.17955)
Supplement: Supplementary file 1 — Table S1. [file BJO-132-178-s001.zip › bjo17955-sup-0002-TableS2.docx]

**Supplementary Table 2: Citations used in coding diagnoses and procedures**

| **Task supported by evidence** | **Reference** |
| --- | --- |
| **Coding of diagnoses and procedures of readmissions relating to pregnancy or childbirth** | Atladottir HO, Thorsen P, Ostergaard L, Schendel DE, Lemcke S, Abdallah M, et al. Maternal infection requiring hospitalization during pregnancy and autism spectrum disorders. J Autism Dev Disord. 2010;40(12):1423-30. |
|  | Blomberg M. Maternal obesity and risk of postpartum hemorrhage. Obstet Gynecol. 2011;118(3):561-8. |
|  | Chen Y, Tang Y, Allen V, DeVivo MJ. Aging and Spinal Cord Injury: External Causes of Injury and Implications for Prevention. Topics in spinal cord injury rehabilitation. 2015;21(3):218-26 |
|  | Conde-Agudelo A, Belizan JM, Lammers C. Maternal-perinatal morbidity and mortality associated with adolescent pregnancy in Latin America: Cross-sectional study. Am J Obstet Gynecol. 2005;192(2):342-9. |
|  | Curran EA, Khashan AS, Dalman C, Kenny LC, Cryan JF, Dinan TG, et al. Obstetric mode of delivery and attention-deficit/hyperactivity disorder: a sibling-matched study. Int J Epidemiol. 2016;45(2):532-42. |
|  | Gulmezoglu AM, Say L, Betran AP, Villar J, Piaggio G. WHO systematic review of maternal mortality and morbidity: methodological issues and challenges. BMC Med Res Methodol. 2004;4:16. |
|  | Harlow BL, Vitonis AF, Sparen P, Cnattingius S, Joffe H, Hultman CM. Incidence of hospitalization for postpartum psychotic and bipolar episodes in women with and without prior prepregnancy or prenatal psychiatric hospitalizations. Arch Gen Psychiatry. 2007;64(1):42-8. |
|  | Jentzsch T, Neuhaus V, Seifert B, Osterhoff G, Simmen HP, Werner CM, et al. The impact of public versus private insurance on trauma patients. J Surg Res. 2016;200(1):236-41. |
|  | Knight M, Callaghan WM, Berg C, Alexander S, Bouvier-Colle MH, Ford JB, et al. Trends in postpartum hemorrhage in high resource countries: a review and recommendations from the International Postpartum Hemorrhage Collaborative Group. BMC Pregnancy Childbirth. 2009;9:55. |
|  | Lambe M, Johansson AL, Altman D, Eloranta S. Mastitis and the risk of breast cancer. Epidemiology. 2009;20(5):747-51. |
|  | Leth RA, Norgaard M, Uldbjerg N, Thomsen RW, Moller JK. Surveillance of selected post-caesarean infections based on electronic registries: validation study including post-discharge infections. J Hosp Infect. 2010;75(3):200-4. |
|  | Mehrabadi A, Liu S, Bartholomew S, Hutcheon JA, Magee LA, Kramer MS, et al. Hypertensive disorders of pregnancy and the recent increase in obstetric acute renal failure in Canada: population based retrospective cohort study. BMJ. 2014;349:g4731. |
|  | Nager A, Sundquist K, Ramirez-Leon V, Johansson LM. Obstetric complications and postpartum psychosis: a follow-up study of 1.1 million first-time mothers between 1975 and 2003 in Sweden. Acta Psychiatr Scand. 2008;117(1):12-9. |
|  | Nolte E, McKee M. Variations in amenable mortality--trends in 16 high-income nations. Health Policy. 2011;103(1):47-52. |
|  | Onwere C, Gurol-Urganci I, Cromwell DA, Mahmood TA, Templeton A, van der Meulen JH. Maternal morbidity associated with placenta praevia among women who had elective caesarean section. Eur J Obstet Gynecol Reprod Biol. 2011;159(1):62-6. |
|  | Palmer WL, Bottle A, Aylin P. Association between day of delivery and obstetric outcomes: observational study. BMJ. 2015;351:h5774. |
|  | Simpson EL, Lawrenson RA, Nightingale AL, Farmer RD. Venous thromboembolism in pregnancy and the puerperium: incidence and additional risk factors from a London perinatal database. BJOG. 2001;108(1):56-60. |
|  | Stanirowski PJ, Bizon M, Cendrowski K, Sawicki W. Randomized Controlled Trial Evaluating Dialkylcarbamoyl Chloride Impregnated Dressings for the Prevention of Surgical Site Infections in Adult Women Undergoing Cesarean Section. Surg Infect (Larchmt). 2016;17(4):427-35. |
|  | Waghorn GR, Chant DC. Employment restrictions among persons with ICD-10 anxiety disorders: characteristics from a population survey. J Anxiety Disord. 2005;19(6):642-57. |
