# Supplementary material for: Emergency Maternal Hospital Readmissions in the Postnatal Period: A Population‐Based Cohort Study
Source: BJOG. 2024 Sep 18;132(2):178–88. doi: 10.1111/1471-0528.17955 (PMC11625651; doi:10.1111/1471-0528.17955)
Supplement: Supplementary file 1 — Table S1. [file BJO-132-178-s001.zip › bjo17955-sup-0003-TableS3.docx]

|  | **Condition** | **ICD-10 coding** |
| --- | --- | --- |
| **Obstetric risk factors recorded in the birth spell** | Chorioamnionitis | O41.1 |
|  | Gestational hypertension | O13 |
|  | Placenta praevia | O44 |
|  | Antepartum haemorrhage | O46, O71.0 |
|  | Preeclampsia | O14 |
|  | Eclampsia | O15.0, O15.9 |
|  | Other hypertension | O10, O11, O16, |
|  | Diabetes in pregnancy | O24 |
|  | Previous caesarean | O34.2, O75.7 |
|  | Premature rupture of membranes | O42 |
|  | Polyhydramnios | O40 |
|  | Oligohydramnios | O41.0 |
|  | Poor foetal growth | O36.5 |
|  | Failed induction of labour | O61 |
|  | Other malpresentation | O64 |
|  | Shoulder dystocia | O66.0 |
|  | Foetal distress | O68 |
|  | Preterm delivery | O60 |
|  | Perineal laceration | O70 |
|  | Other obstetric trauma | O71, N35.0, R32.1, R32.2 |
|  | Medical misadventure | Y60-Y69, Y70-Y84 |
|  | Puerperal sepsis | O85 |
|  | Postpartum haemorrhage | O72, N93.9 |
|  | Retained products of conception | O73 |
|  | Peritonitis | K65 |
|  | Urinary retention | R33, O90.4 |
|  | Mental health conditions | O99.3, F20, F22-F33, F39, F53.0, F53.1, F53.8, F53.9, F39, F40-F45, F48, |
|  | Intra-partum haemorrhage | O67 |
|  | Anaemia with transfusion | O99.0, D64.9 and one of: Z51.3, X33.2, X33.3, X33.8, X33.9, X34 |
|  | Venous thromboembolism | I26.9, I80.1, I80.2, I81 |
|  | Postnatal wound breakdown | O90.0, O90.1, O90.2, O34.2 |
|  | Inflammatory disease of the uterus | N71 |
|  | Other puerperal infection | O96, O75.3, T81.4 |
|  | Difficulty establishing bowel function | K59.0, R15 |
|  | Other maternal factors not elsewhere categorised | O10-O75 or O85-O99 but not already categorised |
|  | Social factors | Z35.7, Z59, Z60, Z63, Z65 |
|  | Stillbirth | Z371, Z373, Z374, Z376, Z377 |
|  | Pre-existing Lupus | M32 |
|  | Pre-existing heart disease | I05-I09, I201, I208, I209, I25, I27, I35-I39 |
|  | Pre-existing Type 2 diabetes | O241, O242 |
|  | Pre-existing asthma | J45-J46 |
|  | Pre-existing sickle cell disease and thalassaemia | D56-D57 |
|  | Gestational diabetes | O244 |
|  | Pre-existing Type 1 diabetes | D56-D57 |
|  | Pre-existing, unspecified diabetes | O243 |
|  | Smoking | F17, Z720 |
|  | Drug use or dependence | F11-F16, F18-F19 |

**Supplementary Table 3: ICD-10 and OPCS-4 coding for obstetric risk factors**
