# Supplementary material for: Emergency Maternal Hospital Readmissions in the Postnatal Period: A Population‐Based Cohort Study
Source: BJOG. 2024 Sep 18;132(2):178–88. doi: 10.1111/1471-0528.17955 (PMC11625651; doi:10.1111/1471-0528.17955)
Supplement: Supplementary file 1 — Table S1. [file BJO-132-178-s001.zip › bjo17955-sup-0004-TableS4.docx]

**Supplementary Table 4: Citations used in coding diagnoses and procedures**

| **Task supported by evidence** | **Reference** |
| --- | --- |
| **Coding of obstetric risk factors** | Carroll F, Stewart L, Knight H, Cromwell D, Gurol-Urganci I, van der Meulen J. Patterns of maternity care in English NHS trusts 2013/14. London: Royal College of Obstetricians and Gynaecologists; 2016. |
|  | Chen Y, Tang Y, Allen V, DeVivo MJ. Aging and Spinal Cord Injury: External Causes of Injury and Implications for Prevention. Topics in spinal cord injury rehabilitation. 2015;21(3):218-26 |
|  | Conde-Agudelo A, Belizan JM, Lindmark G. Maternal morbidity and mortality associated with multiple gestations. Obstet Gynecol. 2000;95(6 Pt 1):899-904. |
|  | Conde-Agudelo A, Belizan JM, Lammers C. Maternal-perinatal morbidity and mortality associated with adolescent pregnancy in Latin America: Cross-sectional study. Am J Obstet Gynecol. 2005;192(2):342-9. |
|  | Curran EA, Khashan AS, Dalman C, Kenny LC, Cryan JF, Dinan TG, et al. Obstetric mode of delivery and attention-deficit/hyperactivity disorder: a sibling-matched study. Int J Epidemiol. 2016;45(2):532-42. |
|  | Elvander C, Ekeus C, Gemzell-Danielsson K, Cnattingius S. Reason for the increasing use of vacuum extraction in Sweden: a population-based study. Acta Obstet Gynecol Scand. 2013;92(10):1175-82. |
|  | Gulmezoglu AM, Say L, Betran AP, Villar J, Piaggio G. WHO systematic review of maternal mortality and morbidity: methodological issues and challenges. BMC Med Res Methodol. 2004;4:16. |
|  | Harlow BL, Vitonis AF, Sparen P, Cnattingius S, Joffe H, Hultman CM. Incidence of hospitalization for postpartum psychotic and bipolar episodes in women with and without prior prepregnancy or prenatal psychiatric hospitalizations. Arch Gen Psychiatry. 2007;64(1):42-8. |
|  | Hogan MC, Foreman KJ, Naghavi M, Ahn SY, Wang M, Makela SM, et al. Maternal mortality for 181 countries, 1980-2008: a systematic analysis of progress towards Millennium Development Goal 5. Lancet. 2010;375(9726):1609-23. |
|  | Jentzsch T, Neuhaus V, Seifert B, Osterhoff G, Simmen HP, Werner CM, et al. The impact of public versus private insurance on trauma patients. J Surg Res. 2016;200(1):236-41. |
|  | Lindqvist PG, Erichs K, Molnar C, Gudmundsson S, Dahlin LB. Characteristics and outcome of brachial plexus birth palsy in neonates. Acta Paediatr. 2012;101(6):579-82. |
|  | Knight HE, Gurol-Urganci I, Mahmood TA, Templeton A, Richmond D, van der Meulen JH, et al. Evaluating maternity care using national administrative health datasets: how are statistics affected by the quality of data on method of delivery? BMC Health Serv Res. 2013;12:200. |
|  | Knight M, Callaghan WM, Berg C, Alexander S, Bouvier-Colle MH, Ford JB, et al. Trends in postpartum hemorrhage in high resource countries: a review and recommendations from the International Postpartum Hemorrhage Collaborative Group. BMC Pregnancy Childbirth. 2009;9:55. |
|  | Lutomski JE, Morrison JJ, Lydon-Rochelle MT. Regional variation in obstetrical intervention for hospital birth in the Republic of Ireland, 2005-2009. BMC Pregnancy Childbirth. 2012;12:123. |
|  | Mehrabadi A, Liu S, Bartholomew S, Hutcheon JA, Magee LA, Kramer MS, et al. Hypertensive disorders of pregnancy and the recent increase in obstetric acute renal failure in Canada: population based retrospective cohort study. BMJ. 2014;349:g4731. |
|  | Nager A, Sundquist K, Ramirez-Leon V, Johansson LM. Obstetric complications and postpartum psychosis: a follow-up study of 1.1 million first-time mothers between 1975 and 2003 in Sweden. Acta Psychiatr Scand. 2008;117(1):12-9. |
|  | Nolte E, McKee M. Variations in amenable mortality--trends in 16 high-income nations. Health Policy. 2011;103(1):47-52. |
|  | Palmer WL, Bottle A, Aylin P. Association between day of delivery and obstetric outcomes: observational study. BMJ. 2015;351:h5774. |
|  | Stanirowski PJ, Bizon M, Cendrowski K, Sawicki W. Randomized Controlled Trial Evaluating Dialkylcarbamoyl Chloride Impregnated Dressings for the Prevention of Surgical Site Infections in Adult Women Undergoing Cesarean Section. Surg Infect (Larchmt). 2016;17(4):427-35. |
|  | Waghorn GR, Chant DC. Employment restrictions among persons with ICD-10 anxiety disorders: characteristics from a population survey. J Anxiety Disord. 2005;19(6):642-57. |
