# Supplementary material for: Emergency Maternal Hospital Readmissions in the Postnatal Period: A Population‐Based Cohort Study
Source: BJOG. 2024 Sep 18;132(2):178–88. doi: 10.1111/1471-0528.17955 (PMC11625651; doi:10.1111/1471-0528.17955)
Supplement: Supplementary file 1 — Table S1. [file BJO-132-178-s001.zip › bjo17955-sup-0005-TableS5.docx]

**Supplementary Table 5: Flow diagram of participant selection**

6,206,638

All women giving birth in English NHS hospitals April 2007-March 2017

47

Wrongly coded as maternal

5

Implausibly young age

3,619

Duplicate spells

2,233

Missing discharge dates

3,673

Implausible admission dates

6,192,140

Eligible women giving birth in English NHS hospitals April 2007-March 2017

6,192,145

Women giving birth in English NHS hospitals April 2007-March 2017

6,192,192

Women giving birth in English NHS hospitals April 2007-March 2017

6,195,811

Women giving birth in English NHS hospitals April 2007-March 2017

6,198,044

Women giving birth in English NHS hospitals April 2007-March 2017

6,201,717

Women giving birth in English NHS hospitals April 2007-March 2017

4,921

Implausibly short intervals between births
