# Supplementary material for: Emergency Maternal Hospital Readmissions in the Postnatal Period: A Population‐Based Cohort Study
Source: BJOG. 2024 Sep 18;132(2):178–88. doi: 10.1111/1471-0528.17955 (PMC11625651; doi:10.1111/1471-0528.17955)
Supplement: Supplementary file 1 — Table S1. [file BJO-132-178-s001.zip › bjo17955-sup-0006-TableS6.docx]

**Supplemental Table 6: Characteristics of mothers with different postnatal lengths of stay**

|  | | **Length of perinatal stay** | | | | |
| --- | --- | --- | --- | --- | --- | --- |
| **Maternal characteristics** | | **Discharge on day of birth**  **628017** | **Discharge 1 day after birth**  **1955156** | **Discharge 2 days after birth**  **1575177** | **Discharge 3 days after birth**  **877445** | **Discharge ≥4 days after birth**  **1158345** |
| **Age (years) (mean, sd)** | | 28.9 (5.6) | 28.8 (5.8) | 29.4 (5.9) | 29.6 (5.9) | 29.8 (6.1) |
| **Age group (years) (mode)** | | 25-29 | 25-29 | 30-34 | 30-34 | 30-34 |
| **Year of giving birth**  **(Number of deliveries, %)** | **2007** | 44814 (9.9) | 139363 (30.9) | 110511 (24.5) | 70618 (15.6) | 86319 (19.1) |
|  | **2008** | 59854 (9.8) | 188790 (31.1) | 152981 (25.2) | 92387 (15.2) | 113735 (18.7) |
|  | **2009** | 59094 (9.7) | 189998 (31.0) | 157951 (25.8) | 91484 (14.9) | 113419 (18.5) |
|  | **2010** | 64234 (10.1) | 200654 (31.5) | 166436 (26.1) | 92508 (14.5) | 113505 (17.8) |
|  | **2011** | 66753 (10.5) | 203058 (31.8) | 165989 (26.0) | 89386 (14.0) | 112554 (17.6) |
|  | **2012** | 68477 (10.6) | 205035 (31.7) | 169515 (26.2) | 88568 (13.7) | 115835 (17.9) |
|  | **2013** | 65373 (10.5) | 196928 (32.0) | 160691 (25.8) | 84888 (13.6) | 114696 (18.4) |
|  | **2014** | 64045 (10.4) | 194546 (31.7) | 155875 (25.4) | 83400 (13.6) | 116484 (19.0) |
|  | **2015** | 62520 (10.2) | 195854 (31.9) | 152091 (24.8) | 83016 (13.5) | 120399 (19.6) |
|  | **2016** | 59357 (9.8) | 195290 (32.1) | 147428 (24.2) | 82272 (13.5) | 124013 (20.4) |
|  | **2017** | 13496 (9.7) | 45640 (32.8) | 33709 (24.2) | 18918 (13.6) | 27386 (19.7) |
| **Ethnicity**  **(n, %)** | **White / White British** | 485324 (11.1) | 1420432 (32.5) | 1107101 (25.3) | 595556 (13.6) | 765145 (17.5) |
|  | **Asian / Asian British** | 47360 (6.9) | 196629 (28.8) | 177429 (26.0) | 108510 (15.9) | 152527 (22.3) |
|  | **Black /Black British** | 18910 (6.2) | 81511 (26.8) | 75850 (25.0) | 50192 (16.5) | 77600 (25.5) |
|  | **Other** | 13919 (7.8) | 54311 (30.3) | 46879 (26.1) | 27609 (15.4) | 36581 (20.4) |
|  | **Mixed** | 8919 (9.4) | 29183 (30.9) | 23997 (25.4) | 13624 (14.4) | 18863 (19.9) |
| **Income domain quintile of the Index of multiple deprivation**  **(N, %)** | **1 (highest income)** | 168312 (9.7) | 556789 (32.1) | 433540 (25.0) | 243347 (14.0) | 332575 (19.2) |
|  | **2** | 140431 (10.2) | 436021 (31.8) | 343658 (25.1) | 193112 (14.1) | 257215 (18.8) |
|  | **3** | 119655 (10.5) | 358853 (31.6) | 288713 (25.4) | 160218 (14.1) | 209547 (18.4) |
|  | **4** | 101885 (10.5) | 306105 (31.5) | 251679 (25.9) | 137648 (14.2) | 174001 (17.9) |
|  | **5 (lowest income)** | 91778 (10.5) | 274534 (31.3) | 229844 (26.2) | 125864 (14.4) | 154958 (17.7) |
| **Parity**  **(n, %)** | **Primiparous** | 93591 (5.8) | 429624 (26.8) | 426955 (26.7) | 268861 (16.8) | 381826 (23.9) |
|  | **Multiparous** | 336397 (13.3) | 894229 (35.2) | 620130 (24.4) | 306104 (12.1) | 380038 (15.0) |
| **Delivery method**  **(n, %)** | **Spontaneous vaginal** | 610108 (16.0) | 1638738 (43.1) | 829062 (21.8) | 343651 (9.0) | 380976 (10.0) |
|  | **Operative vaginal** | 13093 (1.7) | 186544 (23.8) | 251090 (32.0) | 146493 (18.7) | 187501 (23.9) |
|  | **Breech vaginal** | 2305 (8.9) | 7675 (29.5) | 4990 (19.2) | 3316 (12.8) | 7715 (29.7) |
|  | **Elective caesarean section** | 996 (0.2) | 90027 (14.3) | 292991 (46.5) | 120889 (19.2) | 124785 (19.8) |
|  | **Emergency caesarean section** | 1050 (0.1) | 29165 (3.1) | 190580 (20.4) | 259335 (27.8) | 452001 (48.5) |
|  | ***Other** | 72 (13.0) | 187 (33.8) | 125 (22.6) | 84 (15.2) | 86 (15.5) |
| **Delivery setting (n, %)** | **NHS hospital consultant ward** | 187335 (7.6) | 716597 (29.0) | 667007 (27.0) | 382235 (15.4) | 520968 (21.1) |
|  | **NHS hospital midwife ward** | 136788 (22.1) | 256503 (41.5) | 117638 (19.0) | 55170 (8.9) | 61476 (10.0) |
|  | **NHS hospital GP ward** | 6350 (20.4) | 13579 (43.7) | 5692 (18.3) | 2578 (8.3) | 2906 (9.3) |
|  | **NHS hospital: delivery ward with two of: Consultant/ GP/midwife ward** | 177951 (9.5) | 592761 (31.6) | 489569 (26.1) | 268326 (14.3) | 346696 (18.5) |
|  | **NHS hospital ward: no delivery facilities** | 617 (7.8) | 1868 (23.6) | 2114 (26.7) | 1200 (15.1) | 2123 (26.8) |
|  | ****Other** | 7591 (15.7) | 19139 (39.7) | 9812 (20.3) | 5443 (11.3) | 6234 (12.9) |

*destructive operation to facilitate delivery, other specified or other unspecified delivery method

**including private hospital, domestic address followed by admit to hospital, other institution and other setting

Missing data by variable (from a total of 6,192,140); Age 57896, 0.9%; Age group 57896, 0.9%; Year of giving birth 0, 0%; Ethnicity 557909, 9.0%; Income domain of the index of multiple deprivation score 101858, 1.6%; Parity 2054385, 33.2%; Delivery method 16510, 0.3%; Delivery setting 1137874, 18.4%
