# Supplementary material for: Emergency Maternal Hospital Readmissions in the Postnatal Period: A Population‐Based Cohort Study
Source: BJOG. 2024 Sep 18;132(2):178–88. doi: 10.1111/1471-0528.17955 (PMC11625651; doi:10.1111/1471-0528.17955)
Supplement: Supplementary file 1 — Table S1. [file BJO-132-178-s001.zip › bjo17955-sup-0007-TableS7.docx]

| **Ethnicity** | **Income domain of the index of multiple deprivation**  **Mean (sd)** |
| --- | --- |
| **Black / Black British** | 0.26 (0.12) |
| **Asian /Asian British** | 0.24 (0.13) |
| **Other** | 0.22 (0.13) |
| **Mixed** | 0.21 (0.13) |
| **Unknown** | 0.17 (0.12) |
| **White / White British** | 0.16 (0.11) |

**Supplementary Table 7: Mean Income domain on the Index of Multiple Deprivation by Ethnicity**
