# Supplementary material for: Emergency Maternal Hospital Readmissions in the Postnatal Period: A Population‐Based Cohort Study
Source: BJOG. 2024 Sep 18;132(2):178–88. doi: 10.1111/1471-0528.17955 (PMC11625651; doi:10.1111/1471-0528.17955)
Supplement: Supplementary file 1 — Table S1. [file BJO-132-178-s001.zip › bjo17955-sup-0008-TableS8.docx]

**Supplementary Table 8: Maternal characteristics for those readmitted and not readmitted**

|  | | **Emergency maternal readmissions related to pregnancy or childbirth within 42 days of birth (N, %)** | |
| --- | --- | --- | --- |
| **Maternal characteristics** | | **No readmission** | **0-42 days** |
| **Age (years) (mean, sd)** | | 29.3 (5.9) | 29.6 (6.0) |
| **Age group (years) (mode)** | | 30-34 | 30-34 |
| **Year of giving birth**  **(Number of deliveries, %)** | **2007** | 436466 (96.6) | 10912 (2.4) |
|  | **2008** | 587046 (96.6) | 15128 (2.5) |
|  | **2009** | 590221 (93.4) | 15869 (2.6) |
|  | **2010** | 614582 (96.4) | 16928 (2.7) |
|  | **2011** | 613798 (96.2) | 18215 (2.9) |
|  | **2012** | 623529 (96.3) | 18563 (2.9) |
|  | **2013** | 597951 (96.0) | 19199 (3.1) |
|  | **2014** | 589194 (95.9) | 20001 (3.3) |
|  | **2015** | 588672 (95.9) | 19846 (3.2) |
|  | **2016** | 582019 (95.7) | 20734 (3.4) |
|  | **2017** | 133039 (95.6) | 4914 (3.5) |
| **Ethnicity**  **(n, valid %)** | **White / White British** | 4208159 (96.2) | 124374 (2.8) |
|  | **Asian / Asian British** | 655605 (96.0) | 21268 (3.1) |
|  | **Black / Black British** | 288964 (95.0) | 12274 (4.0) |
|  | **Other** | 172216 (96.0) | 5779 (3.2) |
|  | **Mixed** | 90630 (95.8) | 2995 (3.2) |
| **Income domain quintile of the Index of multiple deprivation**  **(N, %)** | **1 (highest income)** | 1664010 (96.0) | 49381 (2.8) |
|  | **2** | 1316854 (96.1) | 40710 (3.0) |
|  | **3** | 1093485 (96.2) | 34350 (3.0) |
|  | **4** | 935990 (96.4) | 28589 (2.9) |
|  | **5 (lowest income)** | 845787 (96.4) | 25887 (3.0) |
| **Parity** | **Primiparous** | 1534151 (95.8) | 52294 (3.3) |
|  | **Multiparous** | 2446410 (96.4) | 67668 (2.7) |
| **Delivery method**  **(n, %)** | **Spontaneous vaginal** | 3689793 (97.0) | 77844 (2.0) |
|  | **Operative vaginal** | 747380 (95.2) | 30904 (3.9) |
|  | **Breech vaginal** | 24709 (95.0) | 836 (3.2) |
|  | **Elective caesarean section** | 601308 (95.5) | 23346 (3.7) |
|  | **Emergency caesarean section** | 876772 (94.1) | 46813 (5.0) |
|  | ***Other** | 524 (94.6) | 25 (4.5) |
| **Delivery setting (n births, valid %)** | **NHS hospital consultant ward** | 2377665 (96.1) | 74255 (3.0) |
|  | **NHS hospital midwife ward** | 598094 (96.8) | 15189 (2.5) |
|  | **NHS hospital GP ward** | 30280 (97.3) | 451 (1.4) |
|  | **NHS hospital: delivery ward with two of: Consultant/ GP/midwife ward** | 1800911 (96.0) | 56484 (3.0) |
|  | **NHS hospital ward: no delivery facilities** | 7573 (95.6) | 285 (3.6) |
|  | ****Other** | 46815 (97.1) | 1025 (2.1) |
| **Obstetric risk factors** | **Chorioamnionitis** | 14601 (93.2) | 834 (5.3) |
|  | **Placenta previa** | 61007 (94.2) | 3041 (4.7) |
|  | **Urinary retention** | 11305 (89.5) | 1214 (9.6) |
|  | **Postnatal wound breakdown** | 8341 (89.6) | 857 (9.2) |
|  | **Venous thromboembolism** | 4263 (90.9) | 343 (7.3) |
|  | **Preeclampsia** | 112360 (91.9) | 8798 (7.2) |
|  | **Stillbirth** | 28301 (91.3) | 1595 (5.1) |
|  | **Other hypertension** | 121450 (93.4) | 7358 (5.7) |
|  | **Gestational hypertension** | 131418 (93.4) | 8032 (5.7) |
|  | **Medical misadventure** | 2979 (91.1) | 249 (7.6) |
|  | **Eclampsia** | 3379 (91.5) | 284 (7.7) |
|  | **Postpartum haemorrhage** | 781729 (94.5) | 38026 (4.6) |
|  | **Pre-existing lupus** | 3013 (93.5) | 176 (5.5) |
|  | **Pre-existing sickle cell disease and thalassaemia** | 44050 (95.8) | 1563 (3.4) |
|  | **Retained products of conception** | 46995 (95.7) | 1630 (3.3) |
|  | **Mental health conditions** | 196803 (94.1) | 8411 (4.0) |
|  | **Preterm delivery** | 470647 (94.5) | 21049 (4.2) |
|  | **Other maternal factors not elsewhere categorised** | 347671 (95.8) | 11329 (3.1) |
|  | **Pre-existing heart disease** | 9009 (94.7) | 399 (4.2) |
|  | **Intra-partum haemorrhage** | 23706 (95.1) | 1033 (41.4) |
|  | **Pre-existing Type 1 Diabetes** | 19193 (93.8) | 970 (4.7) |
|  | **Pre-existing Type 2 Diabetes** | 13759 (93.5) | 761 (5.2) |
|  | **Unspecified Diabetes** | 1301 (93.9) | 70 (5.1) |
|  | **Gestational diabetes** | 215213 (95.4) | 8646 (3.8) |
|  | **Pre-existing asthma** | 346402 (95.3) | 12641 (3.5) |
|  | **Anaemia with transfusion** | 24456 (94.5) | 1091 (4.2) |
|  | **Other obstetric trauma** | 191242 (96.1) | 6254 (3.1) |
|  | **Other puerperal infection** | 61219 (93.8) | 3302 (5.1) |
|  | **Oligohydramnios** | 54314 (95.2) | 2073 (3.6) |
|  | **Polyhydramnios** | 61349 (94.8) | 2650 (4.1) |
|  | **Social factors** | 39309 (94.3) | 1339 (3.2) |
|  | **Previous caesarean** | 606411 (95.5) | 23607 (3.7) |
|  | **Antepartum haemorrhage** | 87374 (95.0) | 3558 (3.9) |
|  | **Perineal laceration** | 2435968 (96.9) | 58180 (2.3) |
|  | **Failed induction of labour** | 672287 (95.0) | 29693 (4.2) |
|  | **Smoking** | 525083 (95.8) | 14229 (2.6) |
|  | **Premature rupture of membranes** | 597543 (96.0) | 19162 (3.1) |
|  | **Puerperal sepsis** | 332028 (94.3) | 15114 (4.3) |
|  | **Poor foetal growth** | 190682 (95.4) | 6526 (3.3) |
|  | **Drug use or dependence** | 20330 (95.2) | 526 (2.5) |
|  | **Shoulder dystocia** | 53097 (96.1) | 1746 (3.2) |
|  | **Other malpresentation** | 122014 (95.1) | 5209 (4.1) |
|  | **Foetal Distress** | 1476810 (95.5) | 56557 (3.7) |
|  | **Peritonitis** | 71 (1.2) | 6 (7.4) |
|  | **Inflammatory disease of the uterus** | 131 (94.2) | 7 (5.0) |
|  | **Difficulty establishing bowel function** | 659 (92.7) | 45 (6.3) |

*destructive operation to facilitate delivery, other specified or other unspecified delivery method

**including private hospital, domestic address followed by admit to hospital, other institution and other setting

Missing data by variable (from a total of 6,192,140): Age 57896, 0.9%; Age group 57896, 0.9%; Year of giving birth 0, 0%; Ethnicity 557909, 9.0%; Income domain of the index of multiple deprivation score 101858, 1.6%; Parity 2054385, 33.2%; Delivery method 16510, 0.3%; Delivery setting 1137874, 18.4%
