# Supplementary material for: Emergency Maternal Hospital Readmissions in the Postnatal Period: A Population‐Based Cohort Study
Source: BJOG. 2024 Sep 18;132(2):178–88. doi: 10.1111/1471-0528.17955 (PMC11625651; doi:10.1111/1471-0528.17955)
Supplement: Supplementary file 1 — Table S1. [file BJO-132-178-s001.zip › bjo17955-sup-0009-TableS9.docx]

**Supplemental Table 9: Characteristics of mothers over time**

| **Maternal characteristics** | | **Year of delivery** | |
| --- | --- | --- | --- |
|  |  | **2008** | **2016** |
| **Age (years) (mean, sd)** | | 28.9 (6.1) | 29.8 (5.6) |
| **Age group (years) (mode)** | | 25-29 | 30-34 |
| **Ethnicity**  **(n, %)** | **White / White British** | 420,382 (77.7) | 421,719 (77.3) |
|  | **Asian / Asian British** | 63,610 (11.8) | 67,255 (12.3) |
|  | **Black /Black British** | 32,141 (5.9) | 26,692 (4.9) |
|  | **Other** | 16,153 (3.0) | 19,952(3.7) |
|  | **Mixed** | 8,698 (1.6) | 9,776 (1.8) |
| **Income domain quintile of the Index of multiple deprivation**  **(N, %)** | **1 (highest income)** | 174,642 (29.0) | 167,038 (28.4) |
|  | **2** | 132,591 (22.0) | 132,718 (22.5) |
|  | **3** | 109,972 (18.3) | 110,759 (18.8) |
|  | **4** | 96,342 (16.0) | 94,278 (16.0) |
|  | **5 (lowest income)** | 89,037 (14.8) | 84,156 (14.3) |
| **Parity**  **(n, %)** | **Primiparous** | 145,811 (44.6) | 138,522 (33.1) |
|  | **Multiparous** | 181,470 (55.4) | 279,788 (66.9) |
| **Delivery method**  **(n, %)** | **Spontaneous vaginal** | 381,741 (63.1) | 363,478 (59.8) |
|  | **Operative vaginal** | 73,726 (12.2) | 77,893 (12.8) |
|  | **Breech vaginal** | 2,546 (0.4) | 2,519 (0.4) |
|  | **Elective caesarean section** | 57,438 (9.5) | 68,138 (11.2) |
|  | **Emergency caesarean section** | 89,659 (14.8) | 95,718 (15.7) |
|  | ***Other** | 44 (<0.1) | 24 (<0.1) |
| **Delivery setting (n, %)** | **NHS hospital consultant ward** | 208,755 (47.4) | 234,179 (46.2) |
|  | **NHS hospital midwife ward** | 35,796 (8.1) | 73,971 (14.6) |
|  | **NHS hospital GP ward** | 7,307 (1.7) | 123 (<0.1) |
|  | **NHS hospital: delivery ward with two of: Consultant/ GP/midwife ward** | 183,654 (41.7) | 192,964 (38.0) |
|  | **NHS hospital ward: no delivery facilities** | 729 (0.2) | 1,548 (0.3) |
|  | ****Other** | 4,329 (1.0) | 4,629 (0.9) |
| **Factors associated with increased readmission risk (%)** | **Urinary retention** | 618 (0.1) | 2,791 (0.5) |
|  | **Postnatal wound breakdown** | 763 (0.1) | 1,199 (0.2) |
|  | **Venous thromboembolism** | 406 (0.1) | 574 (0.1) |
|  | **Preeclampsia** | 11,423 (1.9) | 13,056 (2.1) |
|  | **Stillbirth** | 3,221 (0.5) | 2,741 (0.5) |
|  | **Other hypertension** | 14,087 (2.3) | 10,461 (1.7) |
|  | **Gestational hypertension** | 11,182 (1.8) | 14,990 (2.5) |
|  | **Medical misadventure** | 193 (0.0) | 490 (0.1) |
|  | **Eclampsia** | 432 (0.1) | 303 (0.0)) |
|  | **Postpartum haemorrhage** | 60,860 (10.0) | 106,653 (17.5) |
|  | **Pre-existing lupus** | 190 (0.0) | 467 (0.1) |
|  | **Retained products of conception** | 5,932 (1.0) | 3,781 (0.6) |
|  | **Mental health conditions** | 7,156 (1.2) | 42,051 (6.9) |
|  | **Preterm delivery** | 44,664 (7.3) | 48,681 (8.0) |
|  | **Other maternal factors not elsewhere categorised** | 38,521 (6.3) | 36,492 (6.0) |
|  | **Pre-existing heart disease** | 743 (0.1) | 1,125 (0.2) |
|  | **Intra-partum haemorrhage** | 2,526 (0.4) | 2,617 (0.4) |
|  | **Pre-existing Type 2 Diabetes** | 819 (0.1) | 2,178 (0.4) |
|  | **Pre-existing asthma** | 20,386 (3.4) | 45,774 (7.5) |
|  | **Anaemia with transfusion** | 3,036 (0.5) | 3,256 (0.5) |
|  | **Other obstetric trauma** | 18,057 (3.0) | 17,650 (3.0) |
|  | **Other puerperal infection** | 4,292 (0.7) | 10,719 (1.8) |
|  | **Polyhydramnios** | 3,812 (0.6) | 9,303 (1.5) |
|  | **Social factors** | 1,729 (0.3) | 7,342 (1.2) |
|  | **Previous caesarean** | 51,042 (8.4) | 73,574 (12.1) |
|  | **Gestational diabetes** | 11,949 (2.0) | 35,641 (5.9) |
|  | **Antepartum haemorrhage** | 6,554 (1.1) | 11,903 (2.0) |
|  | **Perineal laceration** | 232,507 (38.3) | 259,715 (42.7) |
|  | **Failed induction of labour** | 57,870 (9.5) | 81,507 (13.4) |

*destructive operation to facilitate delivery, other specified or other unspecified delivery method

**including private hospital, domestic address followed by admit to hospital, other institution and other setting

Missing data by variable (from a total of 6,192,140); Age 57896, 0.9%; Age group 57896, 0.9%; Year of giving birth 0, 0%; Ethnicity 557909, 9.0%; Income domain of the index of multiple deprivation score 101858, 1.6%; Parity 2054385, 33.2%; Delivery method 16510, 0.3%; Delivery setting 1137874, 18.4%
