# Supplementary material for: Emergency Maternal Hospital Readmissions in the Postnatal Period: A Population‐Based Cohort Study
Source: BJOG. 2024 Sep 18;132(2):178–88. doi: 10.1111/1471-0528.17955 (PMC11625651; doi:10.1111/1471-0528.17955)
Supplement: Supplementary file 1 — Table S1. [file BJO-132-178-s001.zip › bjo17955-sup-0010-TableS10.docx]

**Supplementary Table 10: Association of demographic, birth and obstetric risk factors with emergency maternal readmission ≤42 days after birth relating to pregnancy or childbirth: Logistic regression: unadjusted odds ratios**

|  |  | **Odds ratio** | **95% CI** | | **N** |
| --- | --- | --- | --- | --- | --- |
| **Age Group** | **<20yrs** | 1.04 | 1.01 | 1.06 | 301,644 |
|  | **20-24years (comparator)** |  |  |  | 1,096,955 |
|  | **25-29 years** | 1.02 | 1.00 | 1.03 | 1,717,639 |
|  | **30-34 years** | 1.10 | 1.08 | 1.11 | 1,792,166 |
|  | **35-39 years** | 1.17 | 1.15 | 1.19 | 911,683 |
|  | **40+ years** | 1.38 | 1.34 | 1.41 | 234,157 |
| **Year of delivery** | **2007** | 0.97 | 0.95 | 0.99 | 451,625 |
|  | **2008 (comparator as 2007 is not a full data year)** |  |  |  | 607,747 |
|  | **2009** | 1.04 | 1.02 | 1.07 | 611,946 |
|  | **2010** | 1.07 | 1.05 | 1.09 | 637,337 |
|  | **2011** | 1.15 | 1.13 | 1.18 | 637,740 |
|  | **2012** | 1.16 | 1.13 | 1.18 | 647,430 |
|  | **2013** | 1.25 | 1.22 | 1.27 | 622,576 |
|  | **2014** | 1.32 | 1.29 | 1.35 | 614,350 |
|  | **2015** | 1.31 | 1.28 | 1.34 | 613,880 |
|  | **2016** | 1.38 | 1.35 | 1.41 | 608,360 |
|  | **2017** | 1.43 | 1.39 | 1.48 | 139,149 |
| **Ethnicity** | **White / White British (comparator)** |  |  |  | 4,373,558 |
|  | **Asian / Asian British** | 1.10 | 1.08 | 1.12 | 682,725 |
|  | **Black / Black British** | 1.44 | 1.41 | 1.46 | 304,063 |
|  | **Mixed** | 1.12 | 1.08 | 1.16 | 94,586 |
|  | **Other** | 1.14 | 1.11 | 1.17 | 179,299 |
| **Income domain quintile of the Index of multiple deprivation** | **1 (Comparator, highest income)** |  |  |  | 1,734,563 |
|  | **2** | 1.04 | 1.03 | 1.06 | 1,370,437 |
|  | **3** | 1.06 | 1.05 | 1.08 | 1,136,986 |
|  | **4** | 1.03 | 1.02 | 1.05 | 971,318 |
|  | **5** | 1.04 | 1.02 | 1.05 | 876,978 |
| **Parity** | **Primiparity (comparator)** |  |  |  | 1,600,857 |
|  | **Multiparity** | 0.81 | 0.80 | 0.82 | 2,536,898 |
| **Delivery method** | **Spontaneous vaginal delivery (comparator)** |  |  |  | 3,802,535 |
|  | **Operative vaginal delivery** | 1.96 | 1.94 | 1.99 | 784,721 |
|  | **Breech vaginal delivery** | 1.59 | 1.48 | 1.70 | 26,001 |
|  | **Elective caesarean section** | 1.84 | 1.82 | 1.87 | 629,688 |
|  | **Emergency or other type of caesarean section** | 2.53 | 2.50 | 2.56 | 932,131 |
|  | ***Other** | 2.26 | 1.51 | 3.38 | 554 |
| **Place of delivery** | **NHS hospital: delivery facilities with consultant ward (comparator)** |  |  |  | 2,474,142 |
|  | **NHS hospital: delivery facilities with midwife ward** | 0.81 | 0.80 | 0.83 | 617,575 |
|  | **NHS hospital: delivery facilities with GP ward** | 0.48 | 0.43 | 0.52 | 31,105 |
|  | **NHS hospital: delivery facilities with two of: consultant/GP/midwife ward** | 1.00 | 0.99 | 1.01 | 1,875,303 |
|  | **NHS hospital: ward/unit without delivery facilities** | 1.21 | 1.07 | 1.36 | 7,922 |
|  | ****Other** | 0.70 | 0.66 | 0.75 | 48,219 |
| **Length of postnatal hospital stay** | **Discharged on day of birth (comparator)** |  |  |  | 628,017 |
|  | **Discharged day after birth** | 1.26 | 1.23 | 1.28 | 1,955,156 |
|  | **Discharged 2 days after birth** | 1.93 | 1.89 | 1.97 | 1,573,177 |
|  | **Discharged 3 days after birth** | 2.51 | 2.45 | 2.56 | 877,445 |
|  | **Discharged 4 or more days after birth** | 2.92 | 2.86 | 2.99 | 1,158,345 |
| **Obstetric risk factors** | **Urinary retention** | 3.56 | 3.36 | 3.78 | 12,630 |
|  | **Postnatal wound breakdown** | 3.39 | 3.16 | 3.64 | 9,309 |
|  | **Eclampsia** | 2.78 | 2.46 | 3.14 | 3,693 |
|  | **Medical misadventure** | 2.75 | 2.42 | 3.13 | 3,271 |
|  | **Preeclampsia** | 2.67 | 2.61 | 2.73 | 122,260 |
|  | **Peritonitis** | 2.67 | 1.16 | 6.13 | 81 |
|  | **Venous thromboembolism** | 2.63 | 2.36 | 2.94 | 4,690 |
|  | **Difficulty establishing bowel function** | 2.25 | 1.67 | 3.05 | 711 |
|  | **Gestational hypertension** | 2.07 | 2.02 | 2.11 | 140,654 |
|  | **Other hypertension** | 2.04 | 1.99 | 2.09 | 129,970 |
|  | **Pre-existing lupus** | 1.93 | 1.66 | 2.25 | 3,221 |
|  | **Chorioamnionitis** | 1.88 | 1.75 | 2.02 | 15,661 |
|  | **Pre-existing Type 2 Diabetes** | 1.85 | 1.72 | 1.99 | 14,709 |
|  | **Stillbirth** | 1.82 | 1.73 | 1.91 | 31,002 |
|  | **Pre-existing, unspecified diabetes** | 1.81 | 1.42 | 2.30 | 1,385 |
|  | **Other puerperal infection** | 1.79 | 1.73 | 1.85 | 65,289 |
|  | **Inflammatory diseases of the uterus** | 1.77 | 0.83 | 3.78 | 139 |
|  | **Postpartum haemorrhage** | 1.77 | 1.75 | 1.79 | 827,310 |
|  | **Pre-existing Type 1 diabetes** | 1.69 | 1.58 | 1.80 | 20,454 |
|  | **Placenta previa** | 1.65 | 1.59 | 1.72 | 64,734 |
|  | **Failed induction of labour** | 1.55 | 1.53 | 1.57 | 708,039 |
|  | **Puerperal sepsis** | 1.54 | 1.51 | 1.57 | 352,144 |
|  | **Preterm delivery** | 1.53 | 1.51 | 1.56 | 498,203 |
|  | **Anaemia with transfusion** | 1.47 | 1.38 | 1.56 | 25,867 |
|  | **Pre-existing heart disease** | 1.46 | 1.32 | 1.62 | 9,509 |
|  | **Intra-partum haemorrhage** | 1.44 | 1.36 | 1.54 | 24,935 |
|  | **Polyhydramnios** | 1.43 | 1.38 | 1.49 | 64,711 |
|  | **Other malpresentation** | 1.42 | 1.38 | 1.46 | 128,315 |
|  | **Mental health conditions** | 1.42 | 1.38 | 1.45 | 209,174 |
|  | **Foetal distress** | 1.39 | 1.37 | 1.40 | 1,546,771 |
|  | **Gestational diabetes** | 1.35 | 1.32 | 1.38 | 225,680 |
|  | **Antepartum haemorrhage** | 1.35 | 1.30 | 1.39 | 91,984 |
|  | **Previous caesarean** | 1.33 | 1.31 | 1.35 | 635,257 |
|  | **Oligohydraminos** | 1.26 | 1.21 | 1.32 | 57,068 |
|  | **Pre-existing asthma** | 1.22 | 1.19 | 1.24 | 363,403 |
|  | **Pre-existing sickle cell disease and thalassaemia** | 1.17 | 1.08 | 1.26 | 45,981 |
|  | **Retained products of conception** | 1.15 | 1.09 | 1.20 | 49,117 |
|  | **Poor foetal growth** | 1.13 | 1.10 | 1.16 | 199,832 |
|  | **Social factors** | 1.11 | 1.05 | 1.17 | 41,704 |
|  | **Shoulder dystocia** | 1.09 | 1.04 | 1.14 | 55,248 |
|  | **Other obstetric trauma** | 1.08 | 1.06 | 1.11 | 198,999 |
|  | **Other maternal factors not elsewhere categorised** | 1.08 | 1.06 | 1.10 | 362,875 |
|  | **Premature rupture of membranes** | 1.07 | 1.05 | 1.08 | 622,634 |
|  | **Drug use or dependence** | 0.84 | 0.77 | 0.92 | 21,352 |
|  | **Smoking** | 0.88 | 0.86 | 0.89 | 548,295 |
|  | **Perineal laceration** | 0.69 | 0.68 | 0.70 | 2,513,690 |

*destructive operation to facilitate delivery, other specified or other unspecified delivery method

**including private hospital, domestic address followed by admit to hospital, other institution and other setting

Missing data by variable (from a total of 6,192,140): Age group 57896, 0.9%; Year of giving birth 0, 0%; Ethnicity 557909, 9.0%; Income domain of the index of multiple deprivation score 101858, 1.6%; Parity 2054385, 33.2%; Delivery method 16510, 0.3%; Delivery setting 1137874, 18.4%
