# Supplementary material for: Emergency Maternal Hospital Readmissions in the Postnatal Period: A Population‐Based Cohort Study
Source: BJOG. 2024 Sep 18;132(2):178–88. doi: 10.1111/1471-0528.17955 (PMC11625651; doi:10.1111/1471-0528.17955)
Supplement: Supplementary file 1 — Table S1. [file BJO-132-178-s001.zip › bjo17955-sup-0011-TableS11.docx]

| **Non-significant Obstetric risk factors** | **Adjusted odds ratio** | **95% CI** | | **N** |
| --- | --- | --- | --- | --- |
| **Peritonitis** | 2.21 | 0.76 | 6.41 | 81 |
| **Inflammatory diseases of the uterus** | 1.37 | 0.49 | 3.79 | 139 |
| **Pre-existing, unspecified diabetes** | 1.14 | 0.82 | 1.59 | 1,385 |
| **Difficulty establishing bowel function** | 1.11 | 0.72 | 1.70 | 711 |
| **Chorioamnionitis** | 1.06 | 0.96 | 1.16 | 15,661 |
| **Oligohydraminos** | 1.03 | 0.97 | 1.09 | 57,068 |
| **Pre-existing Type 1 diabetes** | 1.03 | 0.94 | 1.12 | 20,454 |
| **Placenta previa** | 1.02 | 0.97 | 1.07 | 64,734 |
| **Shoulder dystocia** | 1.02 | 0.96 | 1.09 | 55,248 |
| **Pre-existing sickle cell disease and thalassaemia** | 1.02 | 0.92 | 1.12 | 45,981 |
| **Foetal distress** | 1.01 | 0.99 | 1.03 | 1,546,771 |
| **Other malpresentation** | 0.99 | 0.95 | 1.03 | 128,315 |

**Supplementary Table 11: Obstetric risk factors not associated with emergency maternal readmission up to 42 days after birth relating to pregnancy or birth (removed from final model)**
